# Clinical and molecular findings in 37 Turkish patients with isolated methylmalonic acidemia

**DOI:** 10.3906/sag-2001-72

**Published:** 2021-06-28

**Authors:** Berna ŞEKER YILMAZ, Deniz KÖR, Fatma Derya BULUT, Sebile KILAVUZ, Serdar CEYLANER, Halise Neslihan ÖNENLİ MUNGAN

**Affiliations:** 1 Department of Pediatric Metabolism, University of Mersin, Faculty of Medicine, Mersin Turkey; 2 Genetics and Genomics Medicine Department, Institute of Child Health, University College London, London UK; 3 Department of Pediatric Metabolism, University Hospital Çukurova, Adana Turkey; 4 Department of Pediatric Metabolism, Adana City Research and Training Hospital, Adana Turkey; 5 Intergen Genetics Laboratories, Ankara Turkey

**Keywords:** Methylmalonic acidemia, novel mutations, complications, outcome

## Abstract

**Background/aim:**

Isolated methylmalonic acidemia (MMA) is caused by complete or partial deficiency of the enzyme methylmalonyl-CoA mutase (mut0 or mut– enzymatic subtype), a defect of its cofactor adenosyl-cobalamin (cblA, cblB, or cblD-MMA), or deficiency of the enzyme methylmalonyl-CoA epimerase. While onset of the disease ranges from the neonatal period to adulthood, most cases present with lethargy, vomiting and ketoacidosis in the early infancy. Major secondary complications are; growth failure, developmental delay, interstitial nephritis with progressive renal failure, basal ganglia injury and cardiomyopathy. We aimed to demonstrate clinical and molecular findings based on long-term follow up in our patient cohort.

**Materials and methods:**

The study includes 37 Turkish patients with isolated MMA who were followed up for long term complications 1 to 14 years. All patients were followed up regularly with clinical, biochemical and dietary monitoring to determine long term complications. Next Generation Sequencing technique was used for mutation screening in five disease-causing genes including; *MUT* , *MMAA* , *MMAB* , *MMADHC* , *MCEE* genes. Mutation screening identified 30 different types of mutations.

**Results:**

While 28 of these mutations were previously reported, one novel MMAA mutation p.H382Pfs*24 (c.1145delA) and one novel MUT mutation IVS3+1G>T(c.752+1G>T) has been reported. The most common clinical complications were growth retardation, renal involvement, mental motor retardation and developmental delay. Furthermore, one of our patients developed cardiomyopathy, another one died because of hepatic failure and one presented with lactic acidosis after linezolid exposure.

**Conclusion:**

We have detected two novel mutations, including one splice-site mutation in the MUT gene and one frame shift mutation in the MMAA gene in 37 Turkish patients. We confirm the genotype-phenotype correlation in the study population according to the long-term complications.

## 1. Introduction 

Isolated methylmalonic acidemia (MMA, OMIM 251000) consists of a group of genetically heterogeneous inborn errors of metabolism characterized by abnormal accumulation of methylmalonyl-CoA and methylmalonic acid (MA) in body fluids without hyperhomocysteinemia [1]. MMA is an autosomal recessive error of organic acid metabolism caused by the impaired isomerization of L-methylmalonyl-CoA to succinylCoA during the oxidation of propionate towards the Krebs cycle [2]. Isolated MMA is caused by complete or partial deficiency of the mitochondrial enzyme L-methylmalonylCoA mutase (MCM, EC 5.4.99.2) (mut 0 enzymatic subtype or mut– enzymatic subtype, respectively), a defect in the handling of its cofactor, AdoCbl (cblA (OMIM607481), cblB (OMIM607568), or cblD2 variant-MMA (OMIM 606169), or deficiency of the enzyme methylmalonyl-CoA epimerase (MCE) [3]. The five genes known to cause isolated MMA include *MUT* , *MMAA* , *MMAB* , *MMADHC* , *MCEE* genes, which are responsible for the MCM, cblA, cblB, and cblD2 variant, MCE deficiency, respectively [4–7]. 

The disease typically presents in the first weeks or months of life and is clinically characterized by recurrent vomiting, poor feeding, failure to thrive, respiratory distress, and neurological deficit from progressive alteration of consciousness to deep coma and death [8]. Ketoacidosis, hypo/hyperglycemia, hyperammonemia, anemia/pancytopenia are the main laboratory findings [8]. Lethal ketoacidosis attacks can follow intercurrent illnesses and it can even mimic diabetic ketoacidosis [9,10]. The mut 0 type, is characterized by significantly low apoenzyme activity, which is not more than 0.1 %, often presents with metabolic acidosis within the first week after birth, frequently resulting in death in early childhood. The mut 0 type usually presents with repeated attacks of ketoacidosis which are triggered by infection and high protein intake during the weaning period after the age of 1 year [11]. 

Methylmalonyl-CoA accumulates in the mitochondrial matrix as a result of MCM deficiency, thus it is subsequently hydrolyzed to CoA and MA, resulting in elevated blood and urine levels of MA [12]. The excessive accumulation of methylmalonic acid and its CoA esters may inhibit mitochondrial enzymes [13,14]. Even with the dietary treatment, affected patients experience various life-threatening metabolic crises, and most patients have problems with growth and motor skills [15]. Although there is a significant improvement in the therapeutic opportunities over the last 20-year-period, the overall outcome of patients with MMA remained almost stable as the number of long-term complications such as failure to thrive, developmental delay, neurologic disorders by degeneration of the basal ganglia, progressive renal failure, and cardiomyopathy has been increasing [16–19]. It is predicted that some mediators like 2-methylcitrate, MA, and other propionate derived mediators inhibit some of the mitochondrial enzymes. 2-methylcitrate inhibits the tricarboxylic acid (TCA) cycle enzymes citrate synthase, aconitase, and isocitrate dehydrogenase.MA inhibits pyruvate carboxylase and propionyl-CoA inhibits CoA dependent enzymes, such as pyruvate dehydrogenase, succinyl-CoA synthetase, and ATP citrate lyase [20]. Furthermore, some studies provided data about the increased levels of lactate particularly in globi pallidi, which indicates a secondary respiratory chain deficiency [14,21]. 

 In this study, we report the clinical features, long term complications, and genetic defects in the *MUT* , *MMAA* , and *MMAB* genes of 37 Turkish patients affected with isolated MMA. 

## 2. Materials and methods 

This is a retrospective cohort study between June 2013 and June 2016. Patient data extracted from previous medical records. Written informed consent obtained from all participants during their clinical visits and conducted according to the Declaration of Helsinki. 

### 2.1. Patients 

The study includes 37 Turkish patients with isolated MMA, who were diagnosed and have been followed up at the Department of Pediatric Metabolism and Nutrition, Çukurova University, Adana, Turkey between June 2013 and June 2016. Unfortunately, none of the patients were diagnosed through neonatal screening, all patients were diagnosed by urine organic acid analysis and the blood carnitine acylcarnitine profile after presenting clinical symptoms and/or developing complications. Enzyme activity assay cannot be performed. Metabolic treatments, including protein restriction (with administration of isoleucine-, methionine-, threonine-, and valine-free special formulas), oral carnitine supplementation, intermittent eradication of gut flora by metronidazole or neomycin, and cobalamin (either oral or intramuscular administration) were given to all patients. They were all followed up for long-term complications 1 to 14 years. Emergency treatment was performed during acute metabolic crises. 

### 2.2. Chronic management and follow-up 

Neurological examination with detailed history of developmental milestones was a routine part of evaluation in every visit to the metabolic clinic. All patients were screened with laboratory markers of renal function including urinary electrolytes and protein loss, blood urea nitrogen (BUN,) and creatinine. Creatinine clearances were calculated according to the Schwartz formula annually in all patients to assess the glomerular filtration rates [22]. A glomerular filtration rate under 80ml/min/1.73 m2 was defined as renal failure. All patients were routinely screened with echocardiography and electrocardiography (ECG) yearly for cardiac complications. 

### 2.3. Mutation analysis 

Mutation analysis performed for all participants and results were obtained from medical records, retrospectively. Genomic DNA was isolated from 2 mL EDTA blood samples obtained by venipuncture on antecubital vein. Next generation sequencing (NGS) technique was used for mutation screening in five disease-causing genes by Illumina-Miseq (Illumina, San Diego, CA, USA) by using in-house designed primers. Mutation analysis was done directly to the gene in question in case there is a preliminary clinical diagnosis. In cases without a differential diagnosis within these five, all five genes were sequenced. The integrative genomics viewer (IGV) software of Broad Institute was used for analysis and comparison with reference sequence. All variations were evaluated by and checked in HGMD-Public version, ClinVar, specific databases, 1000 genome database, EXAC and 2000 exome data of Intergen Genetics Center, Google search for the mutations and all mutations, both previously published and unpublished ones were evaluated with ACMG criterias, DANN, GERP, dbNSFP. FATHMM, FATHMM-MKL, LRT, MetaLR, MetaSVM, Mutation Assessor, Mutation Taster, SIFT, PROVEAN, and Polyphen2. For the splicing defects, we also used Human Splicing Finder program for the prediction. Family screening studies were done for the variants predicted as variant of uncertain significance (VUS) for segregation studies. 

## 3. Results 

A total of 37 patients representing 36 Turkish families (patients 4 and 5 are siblings) are included in this study. Nineteen of 37 (51.4%) patients were male and 18 of 37 (48.6) were female. The clinical features and complementation groups of all patients are summarized in Table 1. Nineteen of 37 patients (51%) presented in the neonatal period (between the ages of 1 and 30 days), while the remaining 18 patients presented in the later infancy period (between the ages of 3 and 24 months). Consanguinity was noted in 33 out of 36 families (92%). The interval from the age of onset to the age at diagnosis was between 2 days and 7 months, except for patient 14, who had an older sibling (patient 13) known to have MMA and was diagnosed at the age of 4 days. Three patients died during subsequent metabolic crises and one of them died because of severe hepatic coma. 32 patients are alive (ages range from 12 months to 14 years), and they were all followed up regularly in the interim period. The most common clinical and biochemical features described were acute episodes of vomiting, poor feeding, failure to thrive, lethargy, hypotonia, and neurological abnormalities with metabolic acidosis, hyperammonemia and methylmalonic aciduria. Other frequently reported features are neurological complications such as developmental delay and mental and motor retardation and renal involvement. 12 out of 37 patients (32%) have some degree of renal involvement. Patient 3 had early-onset and rapidly progressive renal complications, specifically renal tubular acidosis (RTA) type 4 and chronic kidney disease (CKD) stage 3, despite good metabolic control. Patient 10 developed dilated cardiomyopathy with decreased left ventricular ejection fraction (LVEF) of 31% (normal>55%) during a severe metabolic episode at the age of 14 months. When she was 19 months old, she had a severe pneumonia, and she was transferred to the intensive care unit on day 10 of the hospital admission due to the respiratory failure. The antibiotic therapy was advanced to vancomycin. Because of decreased renal function (estimated renal clearance was under 30 mL/min), intravenous linezolid (600 mg q/12 h) was given as an alternative treatment to vancomycin. After the administration of the third dose of linezolid, her blood lactate levels had increased to 10.0 mmol/L. As linezolid was suspected as a potential cause of the lactic acidosis, the agent was stopped. The patient required mechanical ventilation because of respiratory failure and continuous renal replacement therapy was started to normalize her blood pH and clear linezolid from her plasma. 

**Table 1:** Clinical characterization of the isolated MMA patients.

Patientno	Sex	Consan-guinity	Age of onset	Outcome/Current age (years)	Long-term complications	Zygosity	Mutation
1	M	Yes	6 m	Alive/6	DD, GR	H	MMAA gene p.E773* (c.1117G>T)
2	M	Yes	1 m	Alive/8	GR, MMR	H	MUT gene p.R532H (c.1595G>A)
3	M	No	10 m	Alive/15	RI, Liver-renaltransplantation, GR	H	MUT gene p.I671V (c.2011A>G)
4	F	Yes	8 m	Alive/11	DD, MMR, GR	H	MUT gene p.T387I (c.1160C>T)
5	M	Yes	10 m	Alive/6	NA	H	MUT gene p.T387I (c.1160C>T)
6	M	No	12 m	Alive/5	RI, GR	CH	MUT gene c.2055_2056insCTC /p.R727* (c.2179C>T)
7	F	Yes	6 m	Died	MMR, contractures, GR	H	MUT gene p.M1T (c.2T>C)
8	M	Yes	3 d	Alive/6	RI, GR	H	MUT gene p.G454E (c.1361G>A)
9	F	Yes	10 m	Alive/6	DD,RI	H	MMAA gene p.H382Pfs*24 (c.1145delA)
10	F	Yes	3 d	Alive/5	RI,CMP, GR LA	H	MUT gene p.G454E (c.1361G>A)
11	M	Yes	6 m	Alive/12	DD, MMR, RI, GR	H	MMAB gene p.R191W (c.571C>T)
12	F	Yes	20 d	Died	HF, GR	H	MUT gene p.N219Y (c.655A>T)
13	F	Yes	24 m	Alive/8	MMR, RI	H	MMAA gene p.G278D (c.833G>A)
14	F	No	25 d	Died	MMR contractures, GR, RI	H	MUT gene p.K223R (c.668A>G)
15	M	Yes	15 m	Died	MMR, GR, RI	H	MUT gene p.R694W (c.2080C>T)
16	M	Yes	3 d	Alive/3	DD	H	MUT gene p.N219Y (c.655A>T)
17	F	Yes	18 d	Alive/6	MR	H	MMAA gene p.R359Q (c.1076G>A)
18	F	Yes	13 d	Alive/8	RI, DD, MR GR	H	MUT gene p.A141Rfs*39 (c.421delG)
19	F	Yes	3 d	Alive/1	DD	H	MMAA genep.R196* (c.586C>T)
20	F	Yes	22 d	Alive/11	MMR, GR	H	MMAB gene p.E193K (c.577G>A)
21	F	Yes	5 d	Alive/10	GR	H	MMAA gene c.1075C>T (p.R359*)
22	M	Yes	20 d	Alive/1	NA	H	MMAA gene c.658G>A (p.V220M)
23	M	Yes	3 d	Alive/7	DD, RI, GR	H	MUT gene p.347delL(c.1038_1040delTCT)
24	F	Yes	2 d	Alive/5	NA	H	MUT gene p.K121* (c.360_361insT)
25	M	Yes	3 d	Alive/2	NA	H	MMAA gene p.R330*(c.988C>T )
26	F	Yes	4 m	Alive/2	DD	H	MUT gene p.K121* (c.360_361insT)
27	M	Yes	6 m	Died	MMR DystoniaEpilepsy, RI, GR	H	MUT gene p.K121* (c.360_361insT)
28	F	Yes	24 m	Alive/5	NA	H	MMAA gene p.T216P (c.646A>C)
29	F	Yes	16 m	Alive/5	GR	H	MUT gene p.P615T(c.1843C>A )
30	M	Yes	8 m	Alive/4	GR	H	MMAA gene p.R196* (c.586C>T)
31	M	No	1 m	Alive/5	NA	CH	MUT gene p.R108H (c.323G>A)/ IVS3+1G>T (c.752+1G>T)-novel
32	M	Yes	18 m	Alive/6	GR	H	MUT gene p.K121* (c.360_361insT)
33	M	Yes	2 d	Alive/3	NA	H	MUT gene p.N219Y (c.655A>T)
34	M	Yes	2 d	Alive/4	NA	H	MMAB gene p.R186Q (c.557G>A)
35	F	Yes	12 m	Alive/4	NA	H	MMAA gene p.H382Pfs*24 (c.1145delA) -novel
36	M	Yes	29 m	Alive/5	GR	H	MUT gene p.A137G (c.410C>G)
37	F	Yes	2 d	Alive/1	DD	H	MUT genep.R474* (c.1420C>T)

GR: growth retardation; DD: developmental delay; RI: renal involvement; CMP: cardiomyopathy; LA: lactic acidosis; MMR: mental motor retardation; HF: hepatic failure; NA: not available; m:months; d: days; H: homozygous; CH: compound heterozygous.

Eighteen out of thirty-seven patients (49%) had variable neurologic complications, including mental retardation, developmental delay, seizures and metabolic stroke due to the basal ganglia infarction. Growth failure and failure to thrive were identified in 21 out of 37 cases (57%). Overall, 26 out of 37 cases (70%), who are older than 1 year of age, were affected with one of the long-term complications related to isolated MMA. 

Thirty-seven isolated MMA patients were classified in 3 complentation groups: mut, cblA, and cblB. 22 were mut (60%), 12 cblA (32%), and 3 cblB (8%) forms (Table 2). Mutation screening identified 30 mutant alleles in all patients diagnosed as isolated MMA. Thirty-five patients were homozygous, and two patients had compound heterozygous mutations. Ninety-four percent (33/35) of the homozygous patients had documented parental consanguinity, only two were nonconsanguineous. Clinical phenotypes were correlated with the genotypes identified. 

**Table 2:** Identified mutations.

Gene/ Reference Seq	Protein Change	Nucleotid change	Exon	Mutation type	ACMG
MMAA/ NM_172250.2	p.H382Pfs*24	c.1145delA	7	Frame Shift	Pathogenic
MMAA/ NM_172250.2	p.G278D	c.833G>A	6	Missense	VUS
MMAA/ NM_172250.2	p.V220M	c.658G>A	4	Missense	Pathogenic
MMAA/ NM_172250.2	p.E773*	c.1117G>T	7	Nonsense	Pathogenic
MMAA/ NM_172250.2	p.R359Q	c.1076G>A	7	Missense	Pathogenic
MMAA/ NM_172250.2	p.R196*	c.586C>T	4	Nonsense	Pathogenic
MMAA/ NM_172250.2	p.R359*	c.1075C>T	7	Nonsense	Pathogenic
MMAA/ NM_172250.2	p.R330*	c.988C>T	7	Nonsense	Pathogenic
MMAA/ NM_172250.2	p.T216P	c.646A>C	4	Missense	VUS
MMAB/ NM_052845.3	p.R191W	c.571C>T	7	Missense	Pathogenic
MMAB/ NM_052845.3	p.R186Q	c.557G>A	7	Missense	VUS
MMAB/ NM_052845.3	p.E193K	c.577G>A	7	Missense	VUS
MUT/ NM_000255.3	p.R532H	c.1595G>A	9	Missense	Benign
MUT/ NM_000255.3	p.I671V	c.2011A>G	12	Missense	Benign
MUT/ NM_000255.3	p.T387I	c.1160C>T	6	Missense	VUS
MUT/ NM_000255.3	-	c.2055_2056insCTC	12	Deletion-inframe	Likely pathogenic
MUT/ NM_000255.3	p.R727*	c.2179C>T	13	Nonsense	Pathogenic
MUT/ NM_000255.3	p.M1T	c.2T>C	2	Missense	Likely pathogenic
MUT/ NM_000255.3	p.G454E	c.1361G>A	7	Missense	VUS
MUT/ NM_000255.3	p.N219Y	c.655A>T	3	Missense	Likely pathogenic
MUT/ NM_000255.3	p.K223R	c.668A>G	3	Missense	VUS
MUT/ NM_000255.3	p.R694W	c.2080C>T	12	Missense	Likely pathogenic
MUT/ NM_000255.3	p.A141Rfs*39	c.421delG	3	Frame Shift	Pathogenic
MUT/ NM_000255.3	p.K121*	c.360_361insT	2	Nonsense	Pathogenic
MUT/ NM_000255.3	p.P615T	c.1843C>A	11	Missense	Likely pathogenic
MUT/ NM_000255.3	p.R108H	c.323G>A	2	Missense	Pathogenic
MUT/ NM_000255.3	IVS3+1G>T	c.752+1 G>T	3+1	Splicing	Pathogenic
MUT/ NM_000255.3	p.347delL	(c.1038_1040delTCT)	3	Deletion	Likely pathogenic
MUT/ NM_000255.3	p.A137G	c.410C>G	3	Missense	Pathogenic
MUT/ NM_000255.3	p.R474*	c.1420C>T	7	Nonsense	Pathogenic

ACMG: American college of medical genetics; VUS: variant of uncertain significance.

Among identified mutant alleles, 9 different *MMAA* alleles, 3 different *MMAB* alleles, and 18 different *MUT* alleles were described. While 28 of these mutant alleles were previously reported, one novel *MMAA* mutation p.H382Pfs*24 (c.1145delA) and one novel *MUT* mutation IVS3+1G>T (c.752+1G>T) has been reported. p.H382Pfs*24 (c.1145delA) is a frame shift mutation in exon 7, which is detected in both alleles of patient 35. Patient 31, who does not have consanguinity, had IVS3+1G>T (c.752+1G>T), which is located in splice site sequence along with a heterozygous mutated allele p.R108H (c.323G>A). Novel *MUT* and *MMAA* mutations have not been reported in the locus specific databases including, the Human Gene Mutation Database, ClinVar, specific databases, 1000 genome database, EXAC and 2000 exome data of Intergen Genetics Center. 

Two previously recognized polymorphisms in *MUT* gene; p.R532H, and p.I671V were reported in patient 2 and 3, respectively. Both patients presented in the first year of life with vomiting, rapid breathing, and restlessness, and was found to have severe metabolic acidosis with an increased anion gap. Tandem mass spectrometry analysis showed significant elevation of propionylcarnitine, which may be indicative of organic acidemia. While plasma amino acid levels were found to be within normal limits, urine organic acid analysis indicated marked excretion of methylmalonic acid and methylcitric acid, which confirmed the diagnosis of MMA. They both presented with typical MMA features. Under protein-restricted dietary treatment they both had various subsequent metabolic crisis. While patient 2 who had homozygous p.R532H polymorphism developed growth retardation and mental-motor retardation during the follow-up, patient 3 who had p.I671V in a homozygous state presented with severe renal failure and combined liver-renal transplantation was performed at the age of 16. 

## 4. Discussion 

This study expands the mutation spectrum for isolated MMA in the Turkish population particularly highlighting the extent of mutations in the south-eastern part of the country with a large number of patients by using NGS. Moreover, with the classification into the 3 complementation groups, we can slightly anticipate the differences of the phenotypes from the complementation groups and genotypes. 

It is obvious to say that among all of the patients presented in early childhood, almost one half of them had their first symptoms in the neonatal period. All patients are characterized by intermittent metabolic decompensation periods triggered by infections, excessive protein intake, and other stressors. MMA has some major complications such as intellectual impairment, tubulointerstitial nephritis with progressive renal failure, “metabolic stroke” (acute and chronic basal ganglia injury) causing a disabling movement disorder with choreoathetosis, dystonia, and para/quadriparesis; pancreatitis, and growth failure [3]. The most common clinical complications of our patients were growth retardation, renal involvement, mental motor retardation, and developmental delay. Furthermore, one of our patients developed cardiomyopathy, and another one died because of hepatic failure. None of our patients presented with pancreatitis. Moreover, patient 10 presented with life-threatening lactic acidosis after 3 doses of linezolid exposure. While linezolid is increasingly used as a multidrug-resistant antibacterial agent, linezolid-induced lactic acidosis has been frequently reported as a serious side effect [23]. On the other hand, it is commonly believed that MMA causes mitochondrial dysfunction by different mechanisms. While elevated metabolites 2-methylcitrate, methylmalonic acid (MA), and propionyl-CoA inhibit some mitochondrial enzymes, secondary respiratory chain deficiency has also been identified in MMA patients [14]. 2-methylcitrate inhibits citrate synthase, aconitase, and isocitrate dehydrogenase, which are the key enzymes of the tricarboxylic acid cycle (TCA); MA inhibits pyruvate carboxylase, and propionyl-CoA inhibits CoA-dependent enzymes pyruvate dehydrogenase, succinyl-CoA synthetase, and ATP citrate lyase [20]. Thus, neurotoxic metabolites, which inhibit the energy metabolism in various different steps and respiratory chain deficiencies detected in many tissues like liver, muscle, and proximale tubule cells may enhance the formation of lactic acidosis induced by linezolid. 

The *MUT* gene mutations contribute to the majority of the mutant alleles in the Turkish patients, similar to the data from other populations [1,24,25]. We identified a novel splicing mutation c. 752+1G>T (IVS3+1G>T) in one patient in the compound heterozygote state. The second allele was a missense mutation in exon 3; 

c.323G>A (p.R108H) [26]. This patient was presented with metabolic decompensation at the age of 1 month. He is currently alive and 5 years old without any complications under the treatment. 

The frameshift mutation c.360_361insT (p.K121*) was detected in four patients. This single nucleotide insertion causes an immediate stop codon in exon 2. This mutation was first described as a homozygous change in a mut 0 patient who had consanguineous parents [26]. Four of our patients who have this change in a homozygous state presented to the hospital before the age of 2. While three of them are still alive with milder complications, one of them died after developing severe neurological complications and renal failure. 

A missense mutation of c.1160C>T (p.T387I) located in exon 6 was found as a homozygous change in two siblings; patient 4 and 5. Although the older one has renal involvement, developmental delay, and mental retardation as long-term complications, younger one has developed none of these. This mutation has been firstly identified in a 5-year-old Turkish patient who has only mental retardation as a complication [15]. Another missense mutation of c.1361G>A (p.G454E), which is located in the linker region, has been found in two of our patients. Both of them were presented at the third day of life. One of them developed cardiomyopathy and linezolid-induced lactic acidosis besides renal involvement. This missense mutation has been found first in an Italian patient as a heterozygous change with accompanying c.427C>T (p.H143Y) mutation [27]. c.655A>T (p.219Y) mutation is a common missense mutation reported in the Caucasian population particularly in French and Turkish patients [28]. We found this mutation in 3 patients; one of them died because of hepatic failure. 

From presumed missense mutations, we identified two single nucleotide polymorphisms (SNP) p. R532H and p.I69V in the *MUT* gene. Although these changes have been determined as benign, which has not been accepted as a disease-causing variant, both patients had significant clinical and laboratory features of MMA [26]. The functional analysis of this mutant allele bearing both changes will provide insight about the real functional consequences of these sequence variants. Also, while there is no commercial multiplex ligation-dependent probe amplification (MLPA) kit, we could not perform deletion and duplication analysis in these cases. Further molecular analysis should be performed in these patients. 

c.2179C>T (p.R727*), c.2T>C (p.M1T), c. 2055_2056insCTC, c.668A>G (p.K223R), c.2080C>T (p.R694W), c.421delG (p.A141Rfs*39), c.1843C>A (p.P615T), 

c.1038_1040delTCT (p.347delL), c.410C>G (p.A137G) and c.1420C>T (p.R474*) are the other *MUT* mutations, which have been previously reported [15, 26, 27, 29, 30] . 

9 different *MMAA* gene mutations have been described. While c.1145delA (p.H382Pfs*24) is a novel frameshift mutation, other 8 mutations have been reported previously. Four of them were missense mutations. c.833G>A (p.G278D) was identified firstly in an Indian infant who presented at 18 months-old with a typical presentation including ketoacidosis and hyperammonemia [31]. c.658G>A (p.V220M), c.1076G>A (p.R359Q) and c.646A>C (p.T216P) are the other missense MMAA gene mutations which were identified in our patients in a homozygous state. 

4 nonsense previously reported *MMAA* mutations have been identified in our patients. c.586C>T (p.R196*) mutation in exon 4 is a nonsense mutation predicted an amino acid change from arginine to a premature stop codon at position 196 in the mature protein. This nonsense mutation has previously been reported in a compound heterozygote patient with B12-responsive cblA-type methylmalonic academia [32]. It has been also reported in a homozygous state in a Turkish infant mimicking diabetic ketoacidosis [33]. c.1075C>T (p.R359*) is another nonsense mutation in exon 7, which has been previously identified with a high-resolution melting analysis technique [34]. 

c.988C>T (p.R330*) is the other nonsense mutation at codon 330. The mutation is 37 amino acids downstream of a predicted GTP binding site [35]. c.1117G>T (p.E773*) is the fourth nonsense mutation in exon 7, which was found in our patients. 

We have identified 3 missense mutations, which were all previously reported as missense mutations: c.571C>T (p.R191W), c.557G>A (p.R186Q) and c.577G>A (p.E193K) in the *MMAB* gene [4, 36, 37]. 

In conclusion, we have detected two novel mutations, including one splice-site mutation in the *MUT* gene and one frame shift mutation in the *MMAA* gene in 37 Turkish patients. In summary, we have reported the genetic basis of three genes causing isolated MMA in Turkey providing clinical data to explain the phenotypic differences of these disorders. 

## Informed Consent

Written informed consent was taken from the families of participating patients. Çukurova University Medical Faculty Institutional review ethical board approvals for the research project were obtained. Çukurova University Clinical Research Ethics Committee stated that ethical approval was not required as patients were not exposed to a nonroutine practice and an IRB number was not given. The study complied with the World Medical Association Declaration of Helsinki regarding the ethical conduct of research involving human subjects and/or animals.
